# French national diagnosis and care protocol (PNDS) for infantile idiopathic hypercalcemia (IIH)

**DOI:** 10.1186/s13023-026-04483-3

**Published:** 2026-07-17

**Authors:** Cyril Amouroux, Valérie Porquet-Bordes, Elodie Adler, Yoan Le Goff, Justine Bacchetta, Jean Philippe Bertocchio, Gilles Cambonie, Marie Courbebaisse, Odile Dicky, Thomas Edouard, Barbara Girerd, Vincent Guigonis, Fairouz Halitim, Pascal Houiller, Agnès Linglart, Emeline Marquant, Denis Morin, Jean Pierre Salles, Anya Rothenbuhler

**Affiliations:** 1 Endocrinology and Nephrology Unit, Multdisciplinary Paediatrics Department, Arnaud de Villeneuve Hospital, Montpellier, France; 2 Education Departement, School of Medicine, University of Montpellier, Montpellier, France; 3 Endocrine, Bone Diseases and Genetics Unit, Reference Centre for Rare Diseases of Calcium and Phosphate Metabolism, ERN BOND, OSCAR Network, Children’s Hospital, Toulouse University Hospital, RESTORE, INSERM U1301, Toulouse, France; 4 AP-HP, Department of Pediatric Endocrinology, Hôpital Bicêtre Paris Saclay, Le Kremlin- Bicêtre, France; 5 AP-HP, Reference Center for Rare Disorders of the Calcium and Phosphate Metabolism, Filière OSCAR, EndoRare, BOND ERN and Platform of Expertise for Rare Diseases Paris-Saclay, Hôpital Bicêtre Paris Saclay, Le Kremlin- Bicêtre, France; 6 Pathophysiology, Diagnosis and treatments of bone diseases, INSERM UMR1033, Lyon, France; 7 Reference Center for Rare Renal Diseases and Rare Diseases of Phosphate and Calcium, OSCAR and ORKID networks, Hôpital Femme Mère Enfant, Bron, France; 8 AP-HP, Pitié-Salpêtrière Hospital, Thyroid-Endocrine Tumors, Boulevard de l’hôpital, Paris, F–75013 France; 9 Department of Neonatal Medicine, Pediatric Intensive Care and Children’s Acute Transport Service, Arnaud de Villeneuve Hospital, Montpellier University Hospital Centre, University of Montpellier, Montpellier, France; 10 Georges Pompidou European Hospital, Physiology-Metabolic and Renal Functional Explorations Department, AP-HP, Paris Cité University, Reference Center for Rare Calcium and Phosphate Disorders (CaP) and Reference Center for Hereditary Kidney Diseases in Children and Adults (MARHEA), European Rare Kidney Disease Reference Network (ERKNet), Paris, France; 11 Necker-Enfant malades Institut, INSERM U1151, Paris, France; 12 Neonatology and Neonatal Intensive Care Unit, Children’s Hospital, Toulouse University Hospital CHU de Toulouse, Toulouse, France; 13 Department of Pediatrics, Mother and Child Hospital, University Hospital of Limoges, Limoges, France; 14 Private Pediatric Practice, 9 Rue Ernest Cresson, Paris, 75014 France; 15 Centre de Recherche des Cordeliers, INSERM, Sorbonne University, Université Paris Cité, Paris, France; 16 CNRS ERL 8228 - Laboratory of Renal Physiology and Tubulopathies, Paris, France; 17 Endocrine Physiology and Pathophysiology, Université Paris-Saclay, Inserm UMR 1185, Le Kremlin-Bicêtre, France; 18 AP-HM, Multidisciplinary Pediatrics Department, La Timone Children’s Hospital, 264 Rue Saint Pierre, Marseille, 13005 France; 19 CHU Montpellier, Pediatric Nephrology and Endocrinology Department, SORARE Reference Center (Rare Kidney Diseases), Montpellier, 34295 France

**Keywords:** Infantile idiopathic hypercalcemia, Hypercalcemia, Management, Follow-up

## Abstract

The objective of this study was to establish a French National Diagnosis and Care Protocol (PNDS: Protocole National de Diagnostic et de Soins), with the aim of providing health professionals with free, open access synthesis on optimal management and care of patients with Infantile Idiopathic Hypercalcemia (IIH) (https://www.has-sante.fr/jcms/p_3522489/fr/hypercalcemie-infantile-idiopathique-hii). The process involved a critical review of the literature and a multidisciplinary expert consensus. IIH is a rare genetic disorder, with a prevalence estimated at 1 in 33,000. Its clinical manifestations (such as urinary stones or nephrocalcinosis) can appear at different ages. The pathophysiology of IHH is characterized by excessive 1,25(OH)2 vitamin D mediated dietary digestive calcium absorption. The underlying mechanism varies depending on the pathogenic variant involved, which is located in one of two genes, *CYP24A1* or *SLC34A1*. The natural history and pathophysiology of IHH remain poorly understood and are underreported in the literature. Management of the disease varies according to the presentation and severity of hypercalcemia. Non-specific management strategies encompass the cessation of calcium intake, the discontinuation of native vitamin D supplementation, hyperhydration, and the occasional utilization of bisphosphonates. The necessity for long-term treatment, which may encompass both non-specific measures and specific interventions, depends on the severity and the genetic variant identified. Long-term follow-up appears essential, although there is limited data available, particularly for detecting complications of the disease, with nephrocalcinosis being the primary one.

## Background

Idiopathic Infantile Hypercalcemia (IIH) was first described in the 1950s by Lightwood following an epidemic of hypercalcemia cases in infants, secondary to an increase in vitamin D supplementation in infant formulas to prevent rickets in England [1]. Metabolic studies indicated as early as the 1950s that hypercalcemia was secondary to excessive digestive absorption of calcium mediated by vitamin D and that it was associated with hypercalciuria [2]. The hereditary nature and autosomal recessive transmission of IIH were established in 1999, and the two genes involved in IIH, *CYP24A1* and *SLC34A1*, were identified respectively in 2011 and 2016 [3–5]. 

IIH is a rare genetic disease. Its prevalence is estimated at 1/33,000, although this figure may be underestimated due to the underdiagnosis of milder forms. Despite its name, ‘infantile’, clinical manifestations (urinary stones, nephrocalcinosis) can appear or be diagnosed later in childhood or even in adulthood [6].

As mentioned above, there are currently two identified genes:

- The *CYP24A1* gene (MIM *126065), located on 20q13.2, encodes the enzyme 24-hydroxylase. Pathogenic variations in this gene are responsible for type 1 IIH (MIM #143880). This enzyme functions by degrading 25(OH)-vitamin D and 1,25(OH)2-vitamin D (calcitriol, the active form) into 24,25(OH)2-vitamin D and 1,24,25(OH)3- vitamin D (catabolic pathway). A deficiency in the activity of this enzyme results in a decrease in the inactivation of calcitriol [7].
- The *SLC34A1* gene (MIM *182309), located on 5q35.3, encodes the sodium-phosphate co-transporter NaPi-IIa, which is expressed on the apical membrane of proximal renal tubule cells. Pathogenic and biallelic variations in this gene are responsible for type 2 IIH (MIM #616963). The defect in NaPi-IIa membrane expression leads to renal phosphate loss, resulting in a tendency towards hypophosphatemia and inhibition of FGF23 synthesis [5]. This causes both an activation of CYP27B1 expression, leading to increased activity of 1-alpha hydroxylase, and an inhibition of 24-hydroxylase activity.

The underlying pathophysiological mechanism responsible for the occurrence of hypercalcemia in IIH is an excessive digestive absorption of dietary calcium, which is secondary to an elevated concentration of 1,25(OH)2-vitamin D:

- In pathogenic variations of the *CYP24A1* gene, there is a defect in the degradation of 1,25 (OH)2-vitamin D by 24-hydroxylase.
- In pathogenic variations of the *SLC34A1* gene, there is both an increase in the expression of CYP27B1, leading to increased 1-alpha-hydroxylase activity, and an inhibition of 24-hydroxylase activity.

## Objectives and development of the French national diagnosis and care protocol (Protocole National de Diagnostic et de Soin, PNDS)

Numerous healthcare networks are present in France for the management of rare diseases. These networks facilitate patient guidance and care, coordinate research, and develop recommendations for all involved healthcare professionals. The sharing of expertise among these networks leads to the development of recommendations, as evidenced by the current one regarding IIH.

The websites of the networks involved in the creation of this document can be found here: https://maladiesrares-calcium-phosphore.com/ for CRMR CAP (national reference center for rare diseases of calcium and phosphate metabolism) and https://www.filiere-oscar.fr/ for OSCAR (national network for rare bone, calcium, and cartilage diseases).

The process of drafting such recommendations in France is well-defined. These recommendations are established following a thorough review of the existing literature, with the objective of outlining the steps for diagnostic and therapeutic management to harmonize the care and follow-up of patients with IIH across the country. These recommendations can also serve as a reference for general practitioners at each stage of patient care, facilitating coordination with specialist doctors.

This PNDS outlines the standard care for a patient with IIH in 2024. The current PNDS was developed in accordance with the “Method for developing a national protocol for diagnosis and care for rare diseases” published by the French National Authority for Health in 2012, which can be found here: https://www.has-sante.fr/upload/docs/application/pdf/2012-12/guide_methodologique_pnds.pdf.

The coordinating network selected a group of experts, each of whom was affiliated with reference networks involved in the management of IIH and across various medical specialties. These experts conducted comprehensive literature reviews within their respective fields and drafted the synthesis document. The data was pooled, analyzed, and revised during multiple meetings involving both the experts and the coordinating team. The final document was subjected to rigorous editing, review, and approval by all co-authors prior to submission to the health authorities for online publication.

### Diagnosis and initial assessment

#### Clinical presentation

The disease manifests considerable heterogeneity in its presentation, severity, and progression [8]. The most common presentation in infants and young children is acute, varying degrees of hypercalcemia, while adults typically present with kidney stones and/or nephrocalcinosis. The clinical manifestations of hypercalcemia can vary according to the age of the patient. In newborns or infants, signs of hypercalcemia are nonspecific, such as fatigue, growth retardation, irritability, constipation, weight loss, anorexia, vomiting, abdominal pain, and hypotonia. In severe cases, the condition can lead to symptoms such as polyuria (loss of Na + and water) and dehydration, which are associated with acute kidney failure, altered consciousness, and cardiac rhythm disturbances (shortening of the QT interval, prolongation of the PR segment). Nephrocalcinosis may be present as early as the prenatal period and can be asymptomatic [9]. 

In adults or from adolescence onward, renal involvement predominates with symptoms such as kidney stone-related renal colic and/or nephrocalcinosis secondary to hypercalciuria. These renal manifestations can be accompanied by varying degrees of hypercalcemia symptoms, ranging from mild to severe [10–12]. 

In adolescents, hypercalcemia can also manifest with neuropsychiatric disorders such as mood disturbances, psychotic symptoms, confusion, and episodes of hallucinations [13, 14]. 

During pregnancy, women carrying pathogenic variants may develop severe hypercalcemia due to the physiological increase in production of 1,25 (OH)2-vitamin D [15]. 

#### Biochemical profile

The biochemical profile of IIH typically involves hypercalcemia mediated by 1,25 (OH)2-vitamin D: elevated calcium and hypercalciuria, with suppressed PTH secretion and high or abnormally normal levels of 1,25 (OH)2-vitamin D [12]. In pathogenic variants of *SLC34A1*, hypophosphatemia due to urinary phosphate loss may also be present [16]. It is important to note that hypercalciuria originates from both absorptive and resorptive mechanisms, as increased calcitriol stimulates both calcium absorption in the digestive tract and bone resorption [17]. 

**Fig. 1:**
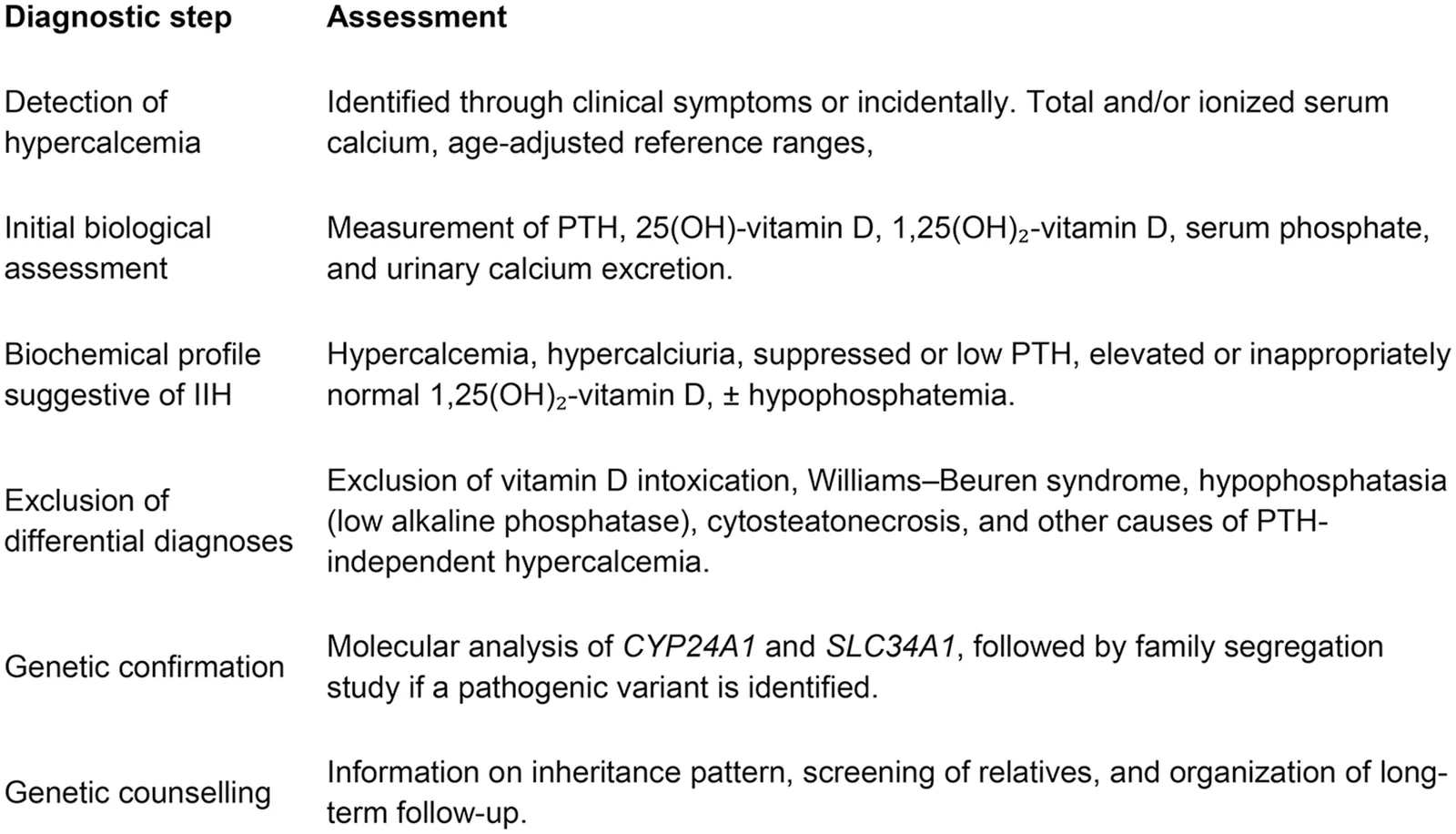
Diagnostic algorithm for infantile idiopathic hypercalcemia (IIH)

The main biological investigations performed as part of the etiological work-up for a phenotype suggestive of IIH are summarized in Table 1, and Fig. 1 illustrates the overall diagnostic algorithm for IIH.

The prescription of biological tests should be tailored according to the context and age of the patient. In the neonatal period, the same assessment can be performed on the parents.

**Table 1:** Main blood and urinary parameters prescribed for a phenotype suggestive of IIH

Blood Tests Best if morning fasting biology	Urinary Tests
Total calcium +/- ionized calcium	*Spot urine collection young children* Calcium, phosphate, creatinine For calculating TmP/GFR and calcium/creatinine ratio
Phosphate	
Magnesium	
ALP	*24-hour Urine Collection in Adolescents and Adults* Calcium excretion Sodium excretion Creatinine Urea Phosphaturia
Creatinine	
Intact PTH 25 (OH)-vitamin D 1,25 (OH)2-vitamin D	

ALP: total alkaline phosphatase, PTH: parathyroid hormone

It is imperative to note that the diagnosis of hypercalcemia can be established only through the meticulous measurement of ionized calcium. The measurement of ionized calcium is a process that necessitates the prompt collection and handling of the sample in a laboratory setting, followed by rapid analysis to ensure the reliability of the interpretation.

In addition, measurement of 24,25(OH)2-vitamin D can be proposed to calculate the ratio of 25 (OH)-vitamin D to 24,25(OH)2-vitamin D, which, when elevated, suggests a pathogenic variant of *CYP24A1*. However, it should be noted that these assays are only performed in specialized laboratories [18, 19]. Hypercalcemia is either identified on a blood test due to symptoms or, on occasion, incidentally during routine testing.

The thresholds delineating hypercalcemia vary between studies and publications. Hypercalcemia is defined in studies as a value above 2.8 mmol/l during the first year of life, above 2.7 mmol/l up to 17 years old, and above 2.6 mmol/l beyond 17 years old. Specific reference ranges are detailed in Table 2 [20, 21]. 

Severe hypercalcemia is traditionally characterized by levels greater than 3.4 mmol/l.

**Table 2:** Norms for serum calcium levels according to age [20, 21].

Reference	Total calcium (mmol/L)	Ionized calcium (mmol/L)
Cord blood	2.05–2.80	1.30–1.60
Neonate (24 h)	NR	1.10–1.36
Neonate (5 days)	NR	1.22–1.38
Birth to 90 days	2.00–2.80	NR
91–180 days	2.20–2.80	NR
181–364 days	2.30–2.80	NR
1–3 years	2.20–2.80	1.20–1.38
4–11 years	2.20–2.70	1.20–1.38
12–18 years	2.10–2.70	1.20–1.38
> 19 years	2.20–2.60	1.16–1.32

NR: not reported

A urinary phosphocalcic assessment is also performed on a sample that includes phosphate, calcium, and creatinine. For adolescents and adults, a 24-hour urine collection is conducted, during which creatinine, urea, sodium, calcium, and phosphate are measured. In children, hypercalciuria is defined as urinary calcium excretion greater than 0.1 mmol/kg/day.

In adults, hypercalciuria is defined as a 24-hour urinary calcium excretion exceeding 6.25 mmol/day in women and 7.5 mmol/day in men.

Specific reference ranges for calcium excretion are provided in Table 3 [22]. 

**Table 3:** Simplified reference values for urinary calcium evaluated by the calcium/creatinine ratio in a morning urine sample [22].

Age range	< 2 years	2–4 years	5–10 years	10–18 years	Adults
Ca/Cr (mmol/mmol)	1.5–2.2	0.7–1.4	0.2–0.8	0.1–0.7	0.05–0.5

Interpretation of calcium excretion in relation to sodium intake and animal protein consumption is important, as excessive intake of these can increase urinary calcium excretion. The reliability of 24-hour urine results is dependent on correct urine collection (neither excessive nor incomplete). The systematic measurement of urinary creatinine ensures the adequacy of the collection. In cases of renal insufficiency, urinary calcium excretion is difficult to interpret (often normal or low).

### Radiologic assessment

A renal imaging study should be performed:

- Renal ultrasound is used to look for nephrocalcinosis and/or kidney stones. It is the reference imaging modality for children.
- In adults, renal computed tomography (CT) complements the evaluation because it is more sensitive for diagnosing nephrocalcinosis and/or kidney stones.

### Evaluation of cardiac involvement by electrocardiography (ECG)

ECG reveal electrophysiological signs of hypercalcemia, including QT interval shortening, flattened or absent T waves, atrioventricular conduction abnormalities, and ventricular arrhythmias.

### Genetics

#### Molecular diagnosis in the index case

The involvement of the genes *CYP24A1* and *SLC34A1* in IIH was demonstrated in 2011 and 2016, respectively [4, 5]. 

A range of molecular analysis techniques (direct sequencing - Sanger method, specific panel testing, exome sequencing, or genome sequencing) are employed, selected based on the context and genetic testing protocols of the specific site.

The objective of these techniques is to study the entire coding regions and intron-exon junctions of the *CYP24A1* and *SLC34A1* genes, with the aim of identifying pathogenic variants responsible for IIH. The most severe forms of IIH are recessive in nature, caused by either two compound heterozygous variants or a homozygous variant [23]. Some patients with a mild form of the disease may carry a single heterozygous variant [24]. Heterozygous patients do not typically present with hypercalcemia but may show hypercalciuria and renal phosphate loss [25]. 

There is also intrafamilial clinical heterogeneity, with phenotypic variability appearing to be correlated with other factors that are currently unknown, potentially involving genetic and environmental influences [3]. 

#### Family segregation study

Following the identification of pathogenic variant(s) in a patient with IIH, family segregation study is essential to identify simple heterozygous carriers with a mild (or even asymptomatic) form of the disease [26]. 

#### Genetic counselling

In the context of genetic analysis for suspected IIH, specialized genetic counselling is available to inform patients (and their families) about the disease and its transmission, provide familial genetic counselling, screen relatives, and support the pregnancy plans of those affected. There is no indication for prenatal diagnosis. Children born into families affected by IIH are screened biologically.

## Differential diagnoses

### Primary differential diagnosis: so-called hypersensitivity to vitamin D

The primary differential diagnosis for IIH is hypersensitivity to vitamin D. Hypersensitivity to vitamin D is more of a transient physiological condition rather than an actual pathology. It is often an incidental finding on blood tests conducted in maternity wards (or within the first few weeks of life), prescribed due to non-specific clinical signs.

The following common features are shared with IIH:

- Hormonal profile: hypercalcemia/hypercalciuria with suppressed PTH and elevated 1,25 (OH)2-vitamin D levels.
- Management: discontinuation of vitamin D supplementation, with very rare indications for specific treatments.

The main differences with IIH are as follows:

- No genetic cause has been identified.
- Average diagnostic serum calcium levels are lower (< 3.4 mmol/L).
- Good clinical tolerance is observed (asymptomatic).
- Favorable evolution occurs in most cases within a few weeks/months.

From a pathophysiological perspective, it is likely due to transient enzymatic immaturity (*CYP24A1*).

### Other causes of hypercalcemia/hypercalciuria with suppressed (or low) PTH in children

#### Renal hypophosphatemic rickets with hypercalciuria (RHH)

The gene *SLC34A3* (MIM *609826), located on 9q34.3, codes for the sodium-phosphate co-transporter NaPi-IIc expressed on the apical membrane of proximal renal tubule cells. Pathogenic biallelic variations in this gene are responsible for RHH (MIM #241530), and the functional consequences are similar to those seen in *SLC34A1*-related disorders. Phenotypically, there is primarily hypophosphatemia due to renal loss independent of FGF23, along with hypercalciuria. Serum calcium levels are generally normal, which distinguishes it from IIH.

In adults, heterozygous mutations in the genes *SLC34A3*, *SLC9A3R1* (encoding NHERF1), and *SLC34A1* are responsible for clinical presentations of primary renal phosphate wasting, which includes renal origin hypophosphatemia, elevated calcitriol levels, and hypercalciuria, but without hypercalcemia.

#### Vitamin D intoxication related to overdose

Vitamin D intoxication can occur due to either prescription errors or accidental excessive intake [27]. There is also a risk from non-medical forms (commonly known as “dietary supplements”) containing Vitamin D that are not regulated as drugs [28]. The biochemical profile includes hypercalcemia, hypercalciuria, suppressed PTH levels, and very high concentrations of 25 (OH)-vitamin D [29]. Severe and symptomatic hypercalcemia may necessitate specific therapeutic interventions.

#### Williams-Beuren syndrome, also known as 7q11 microdeletion syndrome (MIM #194050)

Williams-Beuren syndrome is a condition that affects approximately 1 in 7,500 to 10,000 individuals, characterized by distinctive facial features, learning difficulties, supravalvular aortic stenosis, urinary tract abnormalities, and hypercalcemia with nephrocalcinosis in 5 to 50% of patients [30]. The underlying mechanisms of hypercalcemia, which can self-resolve within days or weeks, remain to be elucidated. However, abnormalities in the metabolism of 1,25 (OH)2-vitamin D and reduced production of calcitonin have been implicated [29, 31].

A PNDS from the AnDDI-Rares network dedicated to this disease is available [32]. 

#### Cytosteatonecrosis

Cytosteatonecrosis typically occurs in the perinatal period in the context of anoxia-ischemia, sepsis, and/or therapeutic hypothermia, and can occur up to the first six weeks of life. It is characterized by the presence of erythematous-violaceous plaques and nodules, which are single or multiple, and are hard to the touch, and can develop into calcifications. These nodules are often found on the back, face, buttocks, and shoulders. This condition is known as acute hypodermatitis. Histologically, it corresponds to lobular panniculitis with fat necrosis and crystallization, granulomatous infiltrate, and calcium deposits.

Hypercalcemia may occur subsequent to the cutaneous manifestations, and its severity is associated with the extent of skin lesions [33]. The hypercalcemia is hypothesized to be dependent on 1,25 (OH)2-vitamin D, with potential macrophage activation of 1-alpha hydroxylase [34]. Therapeutic management is comparable to that of IIH. Monitoring of phosphocalcic balance is essential in this clinical scenario, even though not all cases of cytosteatonecrosis will develop hypercalcemia.

#### Hypophosphatasia

Hypophosphatasia is a rare genetic disease that can present in the neonatal period with simple hypercalcemia/hypercalciuria and low PTH levels [35]. The diagnosis is made on the basis of low or absent serum alkaline phosphatase (ALP) levels, and therefore ALP testing should be considered standard practice in cases of hypercalcemia in pediatrics. Hypophosphatasia primarily affects bone and dental mineralization, and is caused by pathogenic variants in the *ALPL* gene, which codes for tissue-nonspecific alkaline phosphatase [36, 37].

There is a PNDS from the OSCAR network dedicated to this disease [38].

#### Jansen’s metaphyseal chondrodysplasia

It is a rare autosomal dominant disease characterized by short-limbed dwarfism with deformities of the lower limbs, associated with hypercalcemia/hypercalciuria and low PTH levels. It is linked to heterozygous pathogenic variants of the PTH-PTHrP receptor, leading to constitutive activation of the ligand [39].

### Therapeutic management (Fig. 2. Therapeutic algorithm for IHH)

**Fig. 2:**
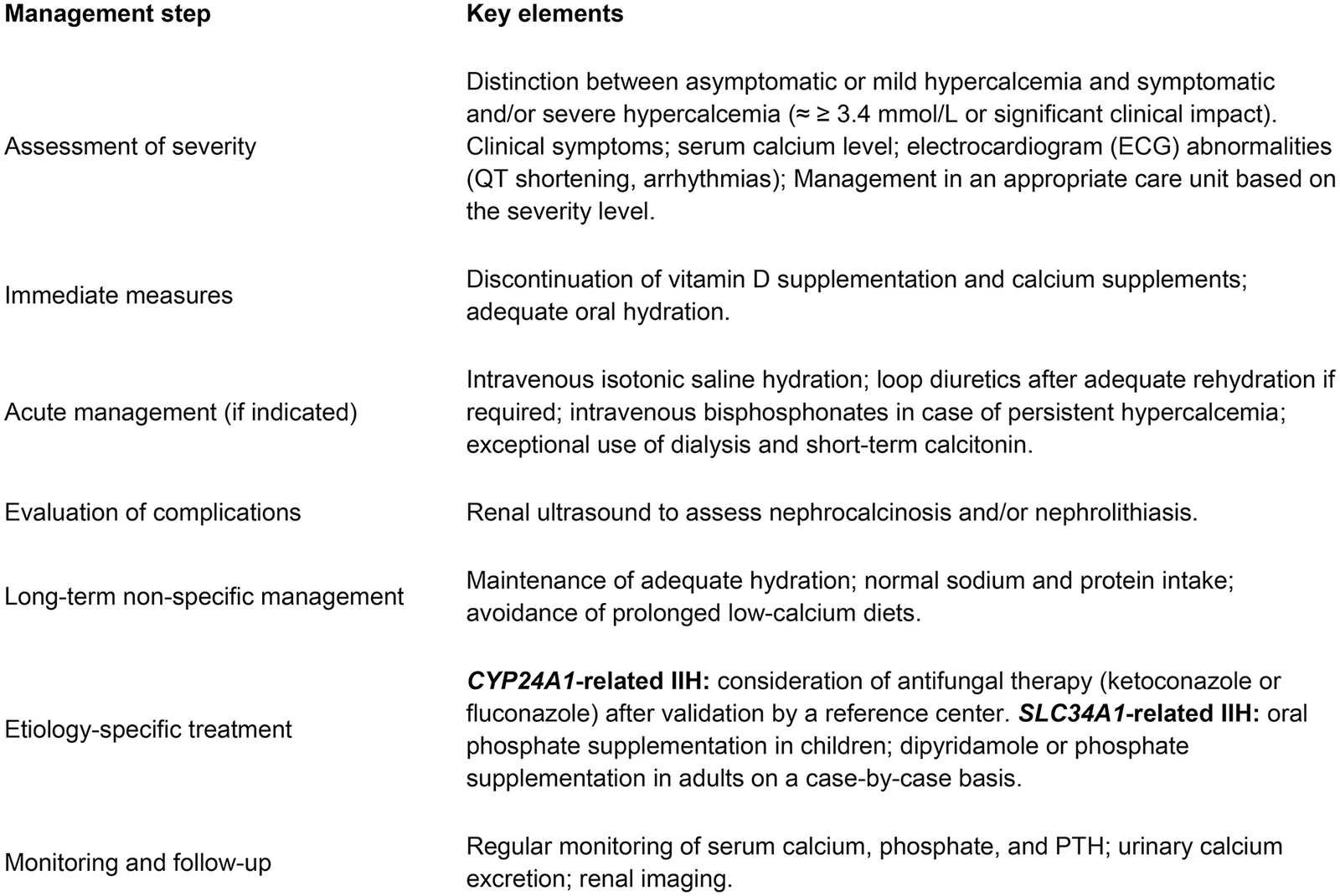
Therapeutic algorithm for infantile idiopathic hypercalcemia (IIH)

#### Emergency treatment of acute hypercalcemia

The indication for emergency treatment depends on the severity of hypercalcemia and, above all, its clinical impact. It should be noted that not all cases of hypercalcemia require urgent therapeutic intervention. There is no formal classification or grading of hypercalcemia severity. However, the severity of clinical signs is often correlated with serum calcium levels and/or the rate of change. Generally, hypercalcemia is considered severe when serum calcium levels reach 3.4 mmol/L, as this is the threshold at which signs and symptoms become apparent in nearly all patients. Any symptomatic hypercalcemia requires urgent management.

The present PNDS aims to delineate the fundamental principles of management strategies without prescribing a rigid protocol.

The following measures are to be considered as primary lines of action: [40]

- Management in an appropriate care unit based on the severity level.
- Discontinuation of calcium intake and hypercalcemic medications (vitamin D supplementation and calcium supplements).
- Restoration of blood volume expansion and rehydration. Isotonic saline (0.9% NaCl) is the most common solution used for this purpose. In addition to expanding volume, rehydration helps reduce serum calcium levels through dilution and increases urinary calcium excretion at the proximal tubule and loop of Henle levels. It is also important to carefully monitor potassium intake due to the potential risk of hypokalemia.
- If these initial measures prove ineffective in lowering serum calcium, the use of furosemide may be considered to further increase calcium excretion, contingent upon prior restoration of fluid volume and careful monitoring thereof [40].

If hypercalcemia persists despite hydration:

- Intravenous bisphosphonate therapy is initiated [40, 41].
- Pamidronate is preferred (dosage between 0.5 and 1 mg/kg depending on age) due to its relatively short half-life. The hypocalcemic effect is observed within 12 to 24 h. A second dose may be administered on a case-by-case basis [42]. The mechanism of action involves the inhibition of bone resorption through the apoptosis of osteoclasts.
- In cases of extremely severe hypercalcemia (exceptionally in cases of IIH), dialysis may be required [40].
- Calcitonin may be used only exceptionally as a short-term adjunctive treatment in selected cases of acute hypercalcemia [40, 43–47]. It inhibits osteoclastic bone resorption and promotes renal calcium excretion, resulting in a rapid but very transient reduction in serum calcium levels. Its use is limited by tachyphylaxis with repeated administration and by safety concerns related to prolonged exposure; therefore, it should not be considered a main therapeutic option but may be used for up to 48 h in combination with intravenous bisphosphonates while awaiting their full effect.

### Long-term treatment for IIH

Long-term treatment is initiated either after emergency management of acute hypercalcemia or immediately when initial hypercalcemia is clinically well tolerated but presents severity criteria that could lead to long-term renal complications (nephrocalcinosis, nephrolithiasis, renal function impairment). The presence of nephrocalcinosis on ultrasound or persistently high calcium excretion are severity criteria warranting specific treatment implementation. In milder presentations of IIH, without symptoms and severity criteria, management is limited to non-specific treatment (discontinuation of vitamin D supplementation and adequate hydration) and clinical-biological monitoring [48].

#### Non-specific management

Aims to reduce intake of 25 (OH)-vitamin D and calcium.

- Discontinue native vitamin D supplementation.
- Ensure age-appropriate oral hydration.
- For severe cases requiring acute hypercalcemia treatment, low-calcium diet (calcium-free milk such as LOCASOL) recommended only for a limited duration and under close supervision.

Aim to limit hypercalciuria

- Normal protein and sodium diet.

#### Specific management

##### Specific treatment for IIH related to the *CYP24A1* gene mutation

Antifungal medications are used.

These are inhibitors of the 1-alpha hydroxylase (and 25-hydroxylase) through non-specific inhibition of mitochondrial cytochrome P450 [49]. They can potentially cause serious side effects (hepatotoxicity, nephrotoxicity). These medications have not been approved for this indication, and there is a lack of large-scale studies that have validated their efficacy and safety. Our recommendations are based on our expertise and the few clinical cases reported in the literature. We recommend that the therapeutic indication be discussed and validated by the reference center network. The prescription of these treatments requires close clinical and laboratory monitoring, and the duration of prescription should be as short as possible. Potential side effects should be clearly explained to the parents [50].

The two molecules used are:

- Ketoconazole: 3 mg/kg/8 h, orally; risk of severe hepatotoxicity with acute liver failure [49, 51].
- Fluconazole: 3 to 12 mg/kg/day, orally; compared to ketoconazole, it has less potent inhibition of 1-alpha hydroxylase but significantly lower toxicity; currently fewer data available than for ketoconazole [52].

##### Specific treatment for IIH related to the *SLC34A1* and *SLC34A3* genes

Although IIH due to mutations in the *SLC34A1* gene is ultimately associated with excessive calcium absorption dependent on 1,25(OH)2-vitamin D, the initial mechanism involves urinary phosphate wasting in the proximal tubule due to reduced membrane expression of NaPi-IIa co-transporters.

#### In children

In the pediatric population, the primary treatment strategy entails phosphate supplementation (40–60 mg/kg/day, administered in four doses, with each dose administered separately from nursing bottle feedings), accompanied by non-specific measures.

#### In adults

In adults, the administration of dipyridamole (PERSANTINE) has been proposed as a potential treatment option to enhance NaPi-IIa expression at the apical membrane [53–55]. However, this approach frequently exhibits poor tolerance and inconsistent effectiveness. The use of phosphate supplementation is infrequent and should be considered on a case-by-case basis, particularly in cases of severe and progressive bone phenotype. However, this should only be initiated after controlling hypercalciuria (with thiazide diuretic therapy) to limit the risk of worsening renal stone disease or nephrocalcinosis.

#### Another therapeutic option

The use of glucocorticoids to treat hypercalcemia in the context of IIH is documented in the literature (for example, Prednisone 0.5-2 mg/kg/day) [56–58]. They act by inhibiting calcium absorption in the gut and by activating *CYP24A1*. However, it is important to note that the response to glucocorticoids is not consistent, and their long-term use can lead to glucocorticoid-related side effects due to excess exposure.

Hypercalcemia typically resolves during the first few months to years of life, and specific treatment can be gradually reduced and discontinued based on the evolution of blood and urine biomarkers. Given the potential for serious side effects, such as hepatotoxicity, it is essential to limit the duration of treatment as much as possible.

Hypercalciuria can persist after the resolution of hypercalcemia, and the continuation of non-specific measures aims to limit urinary calcium excretion, which over the long term can lead to renal complications.

Management of complications.

- Symptomatic hypercalcemia (as mentioned above).
- Nephrocalcinosis and kidney stones: all the non-specific and specific therapies targeting the mechanisms of IIH mentioned above, which aim to limit hypercalcemia and hypercalciuria.

### Follow-up

The data on the follow-up of this condition remain imprecise, with few available and “empirical” data according to centers. Around 50% of children normalize their calciuria by 6 months of age. The main evolving risk determining the follow-up of this condition is the progression of nephrocalcinosis throughout life, potentially leading to chronic kidney disease, sometimes even in pediatric age [59]. Other risks are almost nonexistent.

Due to its relatively mild nature and favorable outcomes in the majority of cases, caution should be exercised to avoid an over-medicalized approach to the follow-up of these patients (excessive investigations, multiple hospitalizations, and initiation of unnecessary therapies).

### When and how to resume vitamin D supplementation?

Criteria vary significantly between teams and long-term studies are necessary.

The resumption of vitamin D supplementation should be initiated when symptoms of “deficiency” manifest in the context of bone metabolism, which is characterized by an unfavorable calcium balance (equivalent to a state of hypocalcemia). This results in the stimulation of bone resorption to maintain optimal blood calcium levels.

The primary indicators of calcipenia include:

- Rise in PTH.
- Undetectable or very low calcium excretion.

It is therefore logical to resume vitamin D supplementation as soon as the PTH reaches the upper end of the normal range, without waiting for it to exceed the upper limit.

The value depends on the assay technique (Table 4).

**Table 4:** Norms for PTH according to measuring techniques

Kit	Technique	Norms (ng/L)
Roche on a Cobas analyzer	Electro-CLIA	15–65
Liaison 1–84 PTH Assay, DiaSorin	CLIA	6.5–36.8
Liaison XL Assay, DiaSorin	CLIA	5–40
Access Intact, Beckman Coulter	CLIA	10–50
Centaur, Siemens, Deerfield	CLIA	17–84

*CLIA: chemi-luminiscence-immuno assay

The resumption modalities should adhere to the recommendations for the age group. Recent French pediatric recommendations were published in March 2022 [60].

The recommended daily intake for children for the general population is 400 to 800 IU, which can be administered as follows:

- 2 drops per day of vitamin D (providing between 600 and 660 IU depending on the formulation) from 0 to 18 years old.
- For children aged 2 years and older, a single 50,000 IU ampoule of vitamin D administered every three months is recommended.

In adults, daily doses are advised to minimize the risk of hypercalciuric episodes.

A subsequent biological reassessment is advised approximately one month after resumption of vitamin D supplementation.

#### Follow-up after diagnosis

##### Severe forms requiring specific treatment

The recommended blood tests, urine tests, and other exams for the follow-up of patients with severe forms requiring specific treatment are summarized in Table 5.

**Table 5:** Recommended blood tests and exams and their frequencies for severe forms requiring specific treatment

	Frequency
**Blood:** Total or ionized calcium / phosphate levels Blood electrolytes, urea / creatinine PTH 25 (OH)-vitamin D AST, ALT (if antifungal treatment is given)	1 month after starting treatment or changing dosage Every 6 months
**Urine: spot urine ** Creatinine, Calcium Urinary pH	1 month after starting treatment or changing dosage Every 6 months
**Renal ultrasound**:	Once a year

The recommended blood tests, urine tests, and other exams for the follow-up of patients who are not severe and do not require specific treatment (i.e., simply stopping vitamin D and providing general instructions) are summarized in Table 6.

**Table 6:** Recommended blood tests and exams and their frequencies for non-severe forms without specific treatment

	Frequency
**Blood:** Total or ionized calcium / phosphate levels Blood electrolytes, urea / creatinine PTH 25 (OH)-vitamin D	Every 3 to 6 months
**Urine: spot urine** Creatinine, Calcium	Every 2 to 3 months
**Renal ultrasound**:	Once a year

#### Follow-up after resuming vitamin D supplementation

The proposed follow-up schedule, summarized in Table 7, is designed to ensure that patients do not experience a recurrence of hypercalcemia or hypercalciuria upon resuming vitamin D supplementation.

**Table 7:** Recommended blood tests and exams and their frequency after resuming of vitamin D supplementation

	Frequency
**Blood:** Total or ionized calcium / phosphate levels Blood electrolytes, urea / creatinine PTH 25 (OH)-vitamin D +/- 1,25 (OH)2-vitamin D	1 month after resuming Then annually for up to 5 years Then at 8–10 years Then at 12–15 years Then at 15–18 years For adults, every 5 years
**Urine: spot urine** Creatinine, Calcium	1 month after resuming Then annually for up to 5 years Then at 8–10 years Then at 12–15 years Then at 15–18 years For adults, every 5 years
**Renal ultrasound**:	Once every 5 years if normal or non-evolving nephrocalcinosis

## Conclusion

This work provides a comprehensive overview of the current state of knowledge regarding the diagnosis, management, and follow-up of IIH in France. It emphasizes the fundamental aspects of managing this pathology and can serve as a valuable reference source for general practitioners and specialists confronted with this condition.

## Data Availability

Data sharing not applicable to this article as no datasets were generated or analyzed during the current study.

## Abbreviations

IIH: Idiopathic Infantile Hypercalcemia
PAI: Individualized Welcome Project (Projet d’Accueil Individualisé)
ALP: Serum Alkaline Phosphatase
PTH: Parathormon
RHH: Hypophosphatemic rickets with hypercalciuria
